# A Comparative Analysis of Absorbance- and Fluorescence-Based 1,3-Diphenylisobenzofuran Assay and Its Application for Evaluating Type II Photosensitization of Flavin Derivatives

**DOI:** 10.3390/ijms27010066

**Published:** 2025-12-20

**Authors:** Minkyoung Kim, Jungil Hong

**Affiliations:** Department of Food Science and Technology, College of Science and Convergence Technology, Seoul Women’s University, Hwarang-ro 621, Nowon-gu, Seoul 01797, Republic of Korea; rlaalsrud080@swu.ac.kr

**Keywords:** 1,3-diphenylisobenzofuran, riboflavin, photosensitivity, singlet oxygen, fluorescence

## Abstract

Singlet oxygen is a type of reactive oxygen species that is typically generated via type II photosensitization reactions. Since 1,3-diphenylisobenzofuran (DPBF), a commonly used chromogenic probe, exhibits peak absorbance at 410 nm for singlet oxygen detection, it severely interferes with blue light irradiation and compounds that absorb in this wavelength region. This study investigated developing and validating a fluorescence-based method using DPBF to quantitatively analyze the type II photosensitizing property of riboflavin (RF) and its heterocyclic flavin derivatives. DPBF fluorescence-based analysis provided more sensitive and practical results than traditional colorimetric methods. It effectively overcomes spectral interference from colored photosensitizers, such as RF and its derivatives, under blue light irradiation (λ peak 447 nm). This method permitted more effective measurement of their activity without interference from their intrinsic color and maintained high linearity and low variation across different sample concentrations, even with short irradiation times. The type II photosensitizing potency of the tested compounds under blue light was consistently ranked as follows: RF > flavin mononucleotide > flavin adenine dinucleotide > lumiflavin > lumichrome. The results suggest that the DPBF fluorescence-based assay is a more effective approach than colorimetric analysis, making it a practical and reproducible tool for assessing the type II photosensitizing properties of diverse compounds. This study also provides a refinement of an existing probe-based assay for relative comparisons under visible light conditions.

## 1. Introduction

Singlet oxygen (^1^O_2_) is an electronically excited form of molecular oxygen. It differs from the ground-state triplet oxygen (^3^O_2_), which has two non-bonding electrons in a parallel spin state. In contrast, the two non-bonding electrons in singlet oxygen are paired with antiparallel spins, resulting in a higher energy configuration [1]. Singlet oxygen is an unstable excited state that has a higher energy level than triplet oxygen. It is classified as a non-radical, non-ionic reactive oxygen species (ROS) characterized by a short lifetime and high reactivity [2,3]. Singlet oxygen has a strong oxidative potential allowing it to directly oxidize biomolecules, including lipids, proteins, and nucleic acids [4]. This high reactivity enables it to interact with a broad range of organic compounds, thereby contributing to cellular damage, aging, and the development of certain diseases [3,4,5]. Furthermore, singlet oxygen is utilized in a variety of chemical and biological applications, including organic synthesis, broad-spectrum anticancer therapies, antimicrobial photodynamic treatments, and intracellular signal transduction [6,7,8,9].

**Figure 1:**
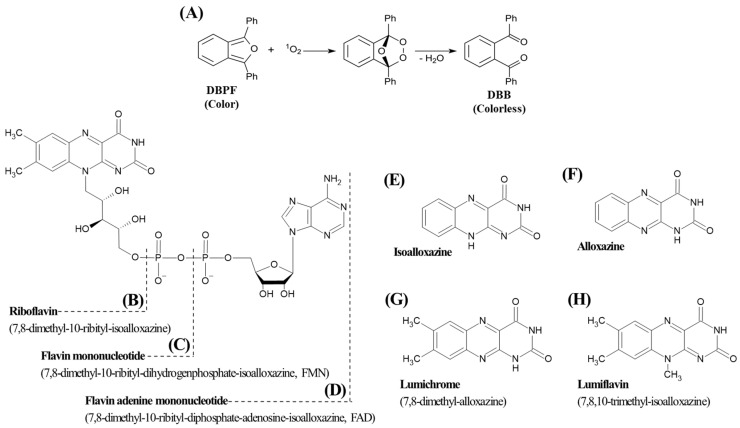
Reaction scheme of 1,3-diphenylisobenzofuran (DPBF) with singlet oxygen (**A**), and structural formula of heterocyclic flavin compounds including, riboflavin (RF) (**B**), flavin mononucleotide (FMN) (**C**), flavin adenine dinucleotide (FAD) (**D**), isoalloxazine (**E**), alloxazine (**F**), lumichrome (LC) (**G**), lumiflavin (LF) (**H**), used in this study.

The detection of singlet oxygen can be performed either directly or indirectly. Direct detection is typically carried out using electron spin resonance (ESR) spectroscopy [10]. Alternatively, indirect detection methods involve the use of chemical probes that selectively react with singlet oxygen. A common probe employed is 1,3-diphenylisobenzofuran (DPBF). It reacts with singlet oxygen to form an unstable endoperoxide intermediate. This intermediate subsequently decomposes into 1,2-dibenzoylbenzene (DBB) (Figure 1A). In contrast to DPBF, which exhibits absorbance, DBB is colorless. Therefore, the extent of singlet oxygen generation can be quantified by monitoring the decrease in absorbance of DPBF [11]. This method offers practical advantages over direct detection, as it can be performed using a conventional UV–visible spectrophotometer without requiring specialized instrumentation for infrared detection.

Photosensitization is defined as the process by which a photosensitizer absorbs light energy and becomes excited. Subsequently, the photosensitizer transfers electrons or energy to surrounding molecules, thereby inducing physical, chemical, or biological changes. Photosensitization can proceed via two distinct pathways: type I and type II. In the type I mechanism, the excited photosensitizer transfers an electron to nearby substrates or molecular species, leading to the generation of radicals. This pathway is capable of occurring independently of the presence of oxygen in the surrounding environment [12]. Reactive species generated via the type I pathway include superoxide radicals (O_2_·^−^), hydroxyl radicals (OH·), alkyl radicals (R·), and lipid peroxyl radicals (ROO·) [12,13]. In contrast, the type II mechanism is oxygen-dependent and involves the transfer of energy from the excited photosensitizer to ground-state molecular oxygen (^3^O_2_), producing singlet oxygen (^1^O_2_) [13,14]. The resulting singlet oxygen can directly oxidize nearby substrates or further react with molecular oxygen to generate secondary ROS [15]. Type I and type II processes may occur simultaneously within the same system, but the dominant pathway is largely influenced by the properties of the photosensitizer, as well as the surrounding environmental conditions and reaction parameters [16].

Riboflavin (7,8-dimethyl-10-(1′-D-ribityl) isoalloxazine, RF) is a water-soluble vitamin that is commonly referred to as vitamin B_2_. It serves as a biochemical precursor of flavin mononucleotide (FMN) and flavin adenine dinucleotide (FAD), all of which share the isoalloxazine ring, a common heterocyclic flavin ring structure (Figure 1B–E) [17]. RF is naturally present in a variety of foods including milk, vegetables, and nuts [18]. While RF is relatively stable to heat, it is sensitive to light [19]. Due to the presence of an isoalloxazine ring moiety, which confers photosensitizing properties, RF is regarded as a representative photosensitizer in foods [17,18,19]. Its derivatives, FMN and FAD, also contain the isoalloxazine structure and have been reported to exhibit similar photosensitizing activity [20]. RF is photodegraded upon exposure to light to form lumichrome (LC) and lumiflavin (LF), which also contain the heterocyclic alloxazine and isoalloxazine structures, respectively (Figure 1E–H). These photodegradation products can also interact with light during food processing, storage, and distribution, potentially causing degradation of nutrients and deterioration of food quality [18,19,21]. Furthermore, they have been implicated in adverse biological effects such as skin aging, dermatitis, and hyperpigmentation [20,21]. Despite these disadvantages, photosensitization has useful applications in fields such as photodynamic therapy (PDT), microbial control, and wastewater treatment, prompting increasing interest in its mechanistic study and potential applications [22,23,24]. To mitigate the disadvantages and maximize the utility of photosensitization, it is essential to first understand the basic functionality and chemical properties of photosensitizers.

Although DPBF has been widely used as a chromogenic probe for singlet oxygen, its photochemical behavior under visible-light irradiation has not been systematically examined. Particularly, the wavelength-dependent instability of DPBF and the potential discrepancies between absorbance- and fluorescence-based detection have not been evaluated in detail, despite their critical importance for accurate quantification. Furthermore, the classical quantum yield measurements reported in the literature are typically obtained using ultraviolet excitation, usually with a laser or lamp emitting below 400 nm. This differs substantially from the visible light environment in which flavins most commonly operate in foods and biological systems. These differences complicate direct application of literature quantum yield values to visible light photoreactions. Therefore, this study aims to comprehensively compare DPBF-based absorbance and fluorescence analyses, investigate the photostability of DPBF under different wavelengths of irradiation, and establish an approach for experimentally validating the type II photosensitization of flavin derivatives under blue-light conditions. This framework allows for accurate and reproducible evaluation of relative photosensitizing potency, independent of quantum yield values obtained under incompatible photophysical conditions.

Importantly, the present study does not aim to determine singlet oxygen quantum yields, elucidate photophysical mechanisms, or introduce a new photochemical probe or metric. Instead, the scope of this work is intentionally limited to the practical and relative comparison of Type II photosensitizing activity under applied visible-light conditions, where classical quantum-yield-based approaches may be unreliable due to probe instability and spectral interference. Accordingly, the DPBF assay is employed here as an application tool for internally consistent, relative comparison, not as a method for absolute photochemical quantification. From this perspective, the present work should be viewed as an incremental and application-focused refinement of an established analytical assay, rather than as a contribution to foundational photochemical theory.

## 2. Results and Discussion

### 2.1. Evaluation of DPBF as a Probe for Type II Photosensitization

**Figure 2:**
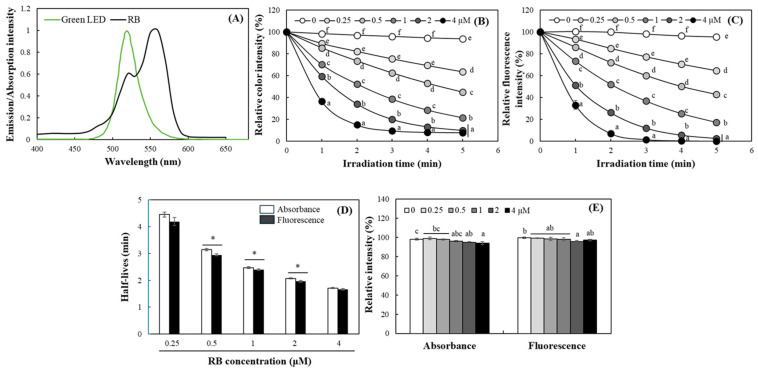
Effects of rose bengal (RB) on color and fluorescence intensities of DPBF under green LED. Emission spectra of green LED and absorbance spectra of RB used in this study were shown (**A**). Changes in absorbance at 410 nm (**B**) and fluorescence at excitation/emission = 350/480 nm (**C**) of DPBF with 0–4 µM RB was monitored under green LED irradiation (1 W/m^2^) during 5 min, and their half-lives were calculated (**D**). Absorbance and fluorescence changes in DPBF stored with 0–4 µM RB in the dark was also measured after 5 min (**E**). Each value represents the mean ± SD (*n* = 3). Different letters indicate a significant difference (*p* < 0.05) based on one way ANOVA and the Tukey’s HSD test. Significantly different between absorbance and fluorescence according to Student’s *t*-test (*, *p* < 0.05 in (**D**)).

In this study, the suitability of 1,3-diphenylisobenzofuran (DPBF) as a probe for sensing type II photosensitizing reaction based on detecting singlet oxygen was evaluated using rose bengal (RB). RB has been reported to generate singlet oxygen under light exposure as a typical type II photosensitizer [25]. Since RB exhibits dual peaks in its UV-vis absorption spectrum, which typically appear ~520 nm and ~560 nm, a green LED emitting within this range was selected as the light source to induce singlet oxygen generation via photosensitization (Figure 2A). The green LED spectrum excites both vibronic absorption bands of RB, with maximal overlap at ~560 nm, enabling efficient triplet-state formation while minimizing direct excitation or photodegradation of the DPBF probe. Thus, although the LED also partially excites the shorter-wavelength RB band, the dominant pathway remains the well-characterized type-II photosensitization mechanism.

RB were exposed to a 1 W/m^2^ green LED during 5 min in the presence of DPBF, and changes in DPBF absorbance were monitored at 410 nm. Color intensity of DPBF decreased with increasing irradiation time and RB concentration. After 3 min of irradiation, the 0.5 μM RB group showed ~55% decrease in absorbance, whereas the 1 μM group showed up to a 76% decrease, demonstrating a clear concentration-dependent effect. For the 1 μM RB group, absorbance decreased by about 14% after 1 min and 55% after 5 min of irradiation (Figure 2B), indicating increased singlet oxygen production over time.

Since DPBF is a fluorescent compound that emits energy in the form of longer wavelengths (450–480 nm) when excited by UVA/blue light [26], the feasibility of a fluorescence-based analytical approach was also assessed. Fluorescence measurements showed that more sensitive changes than absorbance in response to the generation of singlet oxygen by RB exposed to green LED, showing shortened half-lives (Figure 2C,D). In contrast, the color and fluorescence intensity of DPBF was rarely changed under green LED in the absence of RB (Figure 2B,C). No significant changes in absorbance or fluorescence were observed when RB and DPBF were incubated together in the dark for 60 min either (Figure 2E). This consistent trend across both analyses suggests that fluorescence-based detection is also suitable for detecting relative singlet oxygen level via photosensitizing properties. The observed decreases in both absorbance and fluorescence are attributed to the reaction of DPBF with singlet oxygen to form DBB, supporting the validity of fluorescence-based assessment of photosensitizing property.

**Figure 3:**
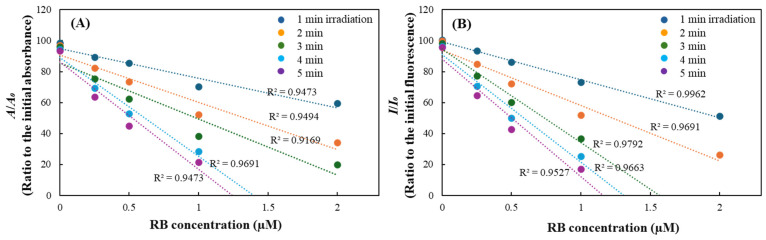
A correlation between absorbance (**A**) or fluorescence (**B**) changes in DPBF and RB concentration at different irradiation periods of blue LED. Each value represents the mean of triplicates.

Subsequently, a linear correlation analysis was conducted between the photosensitizing potency (e.g., singlet oxygen generated) of RB under light irradiation and the changes in the intensity of the color (*A*/*A*_0_, *A*_0_ = initial, *A* = after irradiation) and fluorescence (*I*/*I*_0_, *I*_0_ = initial, *I* = after irradiation) of DPBF. The decolorization and fluorescence reduction response of DPBF exhibited saturation as the RB concentration and irradiation time increased. At a concentration of 4 μM, the color and fluorescence responses of DPBF were saturated within 2 min (Figure 2B,C); these values were excluded from the correlation analysis. The linear correlation coefficient between DPBF decolorization and RB concentration was between 0.91 and 0.97 for irradiation periods of 1 to 5 min (Figure 3A). In contrast, the correlation coefficient with fluorescence changes showed relatively higher correlations ranging from 0.95 to 0.99 over irradiation times of 1 to 5 min. Notably, it exhibited a high correlation within short reaction periods of 1–2 min, demonstrating more sensitive responsiveness than measurements based on colorimetric changes (Figure 3B). These strong correlations confirm that DPBF degradation is directly proportional to relative singlet oxygen level generated. Therefore, both absorbance and fluorescence analysis of DPBF are considered valid and effective approaches for evaluating relative singlet oxygen production via the type II photosensitizing mechanism, and both were employed in subsequent experiments.

### 2.2. Evaluation of DPBF Stability Under Various Light Sources

**Figure 4:**
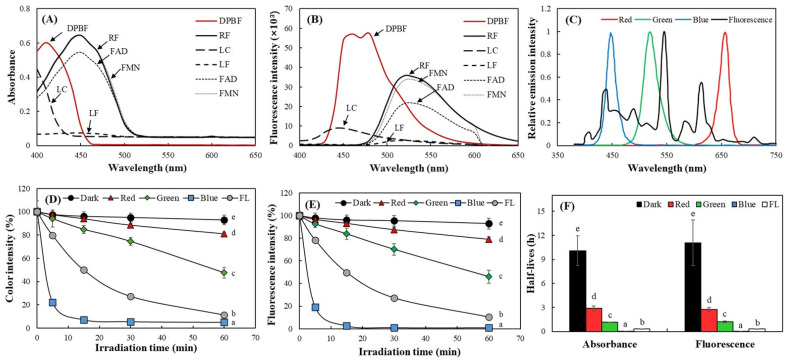
Changes in color and fluorescence intensities of DBPF under different light sources. The spectra profiles of absorbance (**A**) and emission fluorescence upon excitation at 350 nm (**B**) of RF, its derivatives, and DPBF were shown. The light emission profiles of different light sources used in this study including red, green, and blue LEDs and fluorescent light (FL) were also shown (**C**). DPBF was stored in the dark or under the irradiation of different light sources (each 10 W/m^2^), and changes in DPBF intensities of absorbance at 410 nm (**D**) or fluorescence at excitation/emission = 350/480 nm (**E**) were monitored at each time point during 60 min. The half–lives for the absorbance and emission fluorescence of DPBF under the different lights (10 W/m^2^) were also compared (**F**). Each value represents the mean ± SD (*n* = 3). Different letters indicate a significant difference (*p* < 0.05) based on one–way ANOVA and the Tukey’s HSD test.

Photosensitizers activated by light typically have a distinct light absorption spectrum, absorbing light within a specific wavelength range. For instance, RF strongly absorbs light in the 400–450 nm range, overlapping with the light absorption region of DPBF (Figure 4A); this characteristic of certain photosensitizers could severely interfere with DPBF-based singlet oxygen detection generated via their type II reaction. To avoid this limitation, the fluorescence-based measurement of singlet oxygen production from RF and its derivatives was contemplated, and the potential interference of fluorescence in this capacity was investigated. The fluorescence emission spectra of RF and its derivatives were analyzed following excitation at 350 nm, the excitation wavelength for DPBF. The emission fluorescence of DPBF exhibited two peaks near 460 nm and 480 nm. However, most RF derivatives exhibited fluorescence with a peak around 520 nm, whereas only LC showed a peak near 450 nm, similar to the peak observed for DPBF (Figure 4B). The fluorescence intensity of LC was approximately one-sixth that of DPBF, indicating that its interference would be minimal. These findings suggest that for photosensitizers whose light absorption bands overlap with DPBF, fluorescence-based analysis is a more suitable approach than absorbance measurements to minimize spectral interference.

Furthermore, given the substantial influence of the wavelength and intensity of the incident light on the stability of DPBF, the photostability of the probe is a critical factor in the development of effective singlet oxygen detection methods under various lights. Accordingly, DPBF was exposed to different light conditions with various emission wavelengths, including red, green, and blue LEDs, as well as fluorescent light at an intensity of 10 W/m^2^ (Figure 4C). In the dark, DPBF remained relatively stable, with only about 8% decrease in both absorbance and fluorescence signals (Figure 4D,E). Under light conditions, stability varied markedly depending on the light source. After 60 min irradiation, the absorbance of DPBF decreased by 19% and 53% under red and green LEDs, respectively, while substantial decolorization was observed under blue LED and fluorescent light, with absorbance reductions exceeding 90% (Figure 4D). The changes in fluorescence intensity of DPBF showed consistent results, confirming significant photodegradation under blue LED and fluorescent light (Figure 4E). Subsequent comparisons for half-lives of color and fluorescence intensity under different light exposure further supported these observations. Under the irradiation of red and green LEDs, DPBF remained relatively stable with half-lives of 2.9 and 1.1 h based on absorbance, and 2.7 and 1.2 h based on fluorescence, respectively. Conversely, exposure to fluorescent light and blue LED irradiation resulted in a rapid decline in the stability of DPBF, with both the half-lives of color and fluorescence intensity were diminished to less than 2 min under the blue LED (Figure 4F). This phenomenon is likely attributable to the acceleration of DPBF photodegradation, a process that is accompanied by the increased light absorption efficiency of DPBF. This is supported by the observation that the emission wavelength of the blue LED (447 nm) and the emission wavelength of the fluorescent light (410–490 nm) are in close proximity to the maximum absorption wavelength of DPBF (410 nm) (Figure 4C).

The findings indicate that the stability of DPBF is considerably influenced by the emission wavelength of the irradiated light sources. It is noteworthy that high-energy light sources such as blue LED, which emit near the peak absorption of DPBF, can significantly compromise DPBF stability. This may result in substantial interference in the analysis of photosensitizing property using DPBF based on the generation of singlet oxygen. Although the complete avoidance of these light sources is impractical, the modulation of light intensity is considered to be a more viable strategy for ensuring the stability of DPBF within the experimental conditions. In subsequent experiments, the blue LED was retained as the irradiation light source, but its intensity was reduced to minimize photodegradation. This adjustment is expected to suppress DPBF decomposition while preserving sufficient sensitivity for detecting singlet oxygen, thereby potentially enhancing both the precision and reproducibility of the analysis. These results suggest that the wavelength and intensity of the light sources used for irradiation should be carefully controlled when DPBF is used as a probe to detect photosensitization responses. In particular, modulation of the light intensity is essential to ensure reliable and stable measurements in studies using blue LED sources.

### 2.3. Detection of Relative Singlet Oxygen Level Produced by RF and Its Derivatives Using DPBF

It has been established that RF and its derivatives, including FAD and FMN, share an isoalloxazine ring structure that is responsible for their photoreactive properties, exhibiting maximum absorbance at around 450 nm (Figure 4A) [27]. In the subsequent study, a blue LED with a central wavelength of 447 nm was utilized to induce photosensitization in RF and its derivatives. The relative singlet oxygen levels generated by these compounds were subsequently assessed using DPBF as a probe. The preceding results indicate that the irradiation of DPBF with blue LED markedly compromised its photostability.

**Figure 5:**
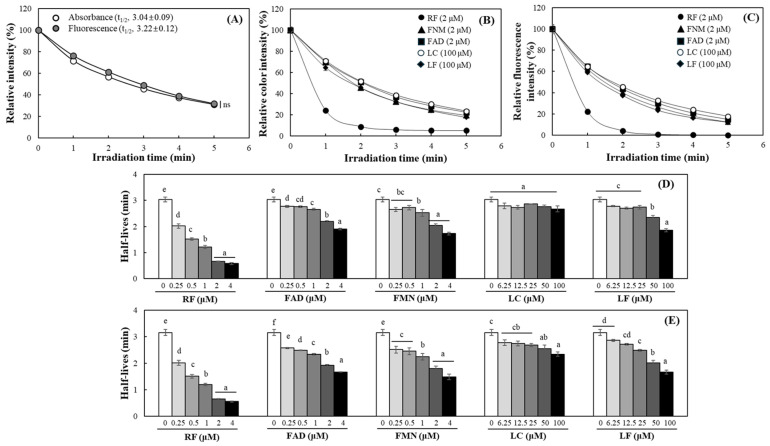
Changes in color and emission fluorescence intensities of DPBF by RF and its derivatives under the irradiation of blue LED. DPBF was irradiated under blue LED (0.5 W/m^2^) and changes in absorbance and fluorescence were monitored during 5 min (**A**). Changes in absorbance at 410 nm (**B**) and fluorescence at excitation/emission = 350/480 nm (**C**) of DPBF in the presence of RF and its derivatives was also monitored under blue LED (0.5 W/m^2^). A comparison was also made of the half–lives of the absorbance (**D**) and emission fluorescence (**E**). Each value represents the mean ± S.D. (*n* = 3). Different letters indicate a significant difference (*p* < 0.05) based on one–way ANOVA and the Tukey’s HSD test.

To address this issue, the experiments were conducted under control with reduced light intensity. DPBF was subjected to blue LED irradiation at 0.5 W/m^2^ for up to 5 min, during which changes in DPBF absorbance and fluorescence were continuously monitored. Even at this reduced light intensity, approximately 80% of DPBF underwent decolorization within 5 min, with no statistically significant differences observed between absorbance- and fluorescence-based measurements showing the extended half-lives of 3.04 ± 0.09 and 3.22 ± 0.12 min, respectively (Figure 5A). The following parameters were adopted for subsequent photosensitization experiments using blue LED irradiation: an intensity of 0.5 W/m^2^ and a maximum irradiation time of 5 min.

RF, FAD, and FMN (each at 2 μM) effectively induced DPBF photobleaching even at 0.5 W/m^2^ of blue LED, while LC and LF only partially decolorized DPBF at much higher concentrations (100 μM) under equal light intensity (Figure 5B). This trend was consistent in both absorbance and fluorescence measurements, with RF exhibiting the highest potency. In contrast, the other compounds showed less potent photobleaching effects over longer irradiation times (Figure 5B,C). An analysis of the half-life of DPBF based on absorbance changes revealed that RF, FAD, and FMN induced concentration-dependent decolorization, even within the 0.25–4 μM concentration range. LF exhibited concentration dependence at much higher ranges of 25–100 μM, whereas LC did not show clear concentration-dependent half-life reduction (Figure 5D). DPBF fluorescence-based analysis demonstrated a heightened sensitivity in detecting photosensitizing property of RF and its derivatives. Especially, LC did not show significant differences in its activity up to 100 μM in absorbance-based measurements; a clear concentration-dependent trend was observed in the fluorescence-based analysis (Figure 5E).

### 2.4. Quantitative Analysis of Photosensitizing Properties of RF and Its Derivatives

The quantitative analysis for the photosensitizing action of RF and its derivatives was performed based on the changes in absorbance and fluorescence of DPBF. Each value was calculated by taking the natural logarithm of DPBF absorbance changes before (A_0_) and after irradiation at each time point (A_t_) [ln (A_0_/A_t_)]. Under constant light intensity, DPBF bleaching by photosensitizers proceeded under pseudo–first-order conditions with respect to DPBF, allowing ln (A_0_/Aₜ) to be used as a quantitative kinetic descriptor. Overall, the calculated values demonstrated a quantitative increase in response to changes in photosensitizer concentration and light exposure time (Table 1). Furthermore, a comparison of the activity among the photosensitizers was facilitated by the values obtained. Both absorbance- and fluorescence-based measurements consistently demonstrated that the photosensitizing potency was highest for RF, followed by FMN, FAD, LF, and LC. In the case of LC, which exhibited the weakest activity and the least change in DPBF response, its activity could only be detected by absorbance-based analysis under irradiation conditions at 100 μM for over 3 min. Conversely, the application of fluorescence-based analysis revealed the activity even at concentrations as low as 6.25 μM following 3 min of irradiation or 25 μM following 1 min of irradiation. RF and its derivatives have been shown to induce more sensitive changes in DPBF responses and exhibit stronger photosensitizing properties. The fluorescence-based analysis has consistently produced higher values than the absorbance-based analysis. It has also demonstrated the capability to detect activity at lower concentrations and shorter reaction times (Table 1).

Notable differences in sensitivity were observed between absorbance- and fluorescence-based detection of relative levels of singlet oxygen generated by RF concentrations of 1 μM or higher under blue LED irradiation. The absorbance-based analysis revealed a decreased response in the measured level after 2 or 3 min of irradiation at these concentration ranges (Table 1, Figure S1A), whereas the fluorescence analysis showed a continuous, linear increase up to 5 min (Figure S1B). A correlation between DPBF response and RF concentration further confirmed the higher sensitivity of the fluorescence-based analysis. The absorbance measurements showed correlation coefficients of 0.745 and 0.931 at 3 and 5 min of irradiation, respectively, while the fluorescence measurements exhibited near-perfect correlation coefficients of 1.000 and 0.998, respectively (Figure S1C). The results indicate that the fluorescence-based analysis is more efficient at detecting a wider range of singlet oxygen levels with greater precision.

### 2.5. Comparison of Photosensitizing Properties of RF and Its Derivatives Under Blue LED

To compare the photosensitizing property through the DPBF responses under light irradiation among RF and its derivatives, it is necessary to normalize their responses under identical concentration or irradiation time conditions. Consequently, the levels of DPBF changes made by photosensitizers of varying concentrations under same light exposure periods (excluding saturated values) was corrected for each concentration, and the mean value was calculated to compare the relative ratios among the photosensitizers (Table 2). The levels of DPBF changes made by each photosensitizer during a 1 min-irradiation showed considerable variation across concentrations. Notably, the adjusted relative DPBF responses among different concentrations at the same time showed a remarkably greater variation and a consistently greater coefficient of variation (CV) when analyzed using the absorbance-based methods compared to fluorescence-based analysis. A consistent value based on reaction time and concentration is essential for stable and precise quantitative analysis. The lower CV values observed in DPBF fluorescence-based analysis suggest that this method is more reliable for evaluating the photosensitivity of RF and its derivatives than the absorbance-based analysis (Table 2).

Normalization of DPBF responses by concentration revealed a consistent increase with irradiation time in all experimental groups, exhibiting a higher correlation coefficient of over 0.99. The relative level (RL) of each RF derivative was calculated by setting the RF-induced DPBF changes during each period of light irradiation to 100. The 1 min reaction value was excluded due to highly variable concentrations. According to the absorbance-based analysis, their activities were exhibited in the following order: FMN (5.10–7.25), FAD (4.15–6.70), LF (0.33–0.42), and LC (0.18–0.26). The relative activity of the derivatives exhibited no change in order during light irradiation but increased continuously over time. The fluorescence-based measurements also revealed the same order of FMN (9.07–10.37), FAD (7.25–7.98), LF (0.40–0.45), and LC (0.25–0.33), but unlike the absorbance measurements, characteristic changes over irradiation time were not observed (Table 2).

Furthermore, the calculation of the slope [d{ln (A_0_/A_t_)/concentration (μM)}/dt] between irradiation time and DPBF responses, which exhibited an exact correlation of 0.99 or higher, revealed the order RF > FMN > FAD > LF > LC in both the absorbance- and fluorescence-based analyses, with more distinct differences observed in the fluorescence analysis. In contrast to the RL of DPBF responses, which varies with irradiation time, this slope serves as a metric enabling quantitative comparison of the activities of RF and its derivatives over the entire reaction time. The relative activity (RA) of each derivative was calculated by establishing the RF that exhibited the strongest potency as 100. The consistent potency of RF, FMN, FAD, LF, and LC was demonstrated by both the absorbance- and fluorescence-based analyses. The relative potency of FMN, FAD, LF, and LC compared to 100 of RF was determined to be 8.398, 8.394, 0.445, and 0.324, respectively, based on the results of the absorbance analysis; the corresponding values derived from the fluorescence-based analysis were 8.591, 6.930, 0.417, and 0.226 (Table 2).

The differences in the photosensitizing properties among RF and its related compounds can be attributed to their distinct structural characteristics. The presence of a flavin isoalloxazine ring is a distinguishing characteristic of RF, FMN, and FAD (Figure 1B–E). This structure confers a notably potent photosensitizing activity in comparison to LC, which is devoid of this particular heterocyclic ring and instead possesses an alloxazine moiety, a tautomer of isoalloxazine (Figure 1F,G). In case of FAD, its adenine moiety establishes a stacking interaction with the flavin ring, thereby facilitating intramolecular electron transfer from the excited flavin to the adenine. This rapid non-radiative deactivation suppresses intersystem crossing, limiting the formation of triplet states and reducing the yield of singlet oxygen in comparison with RF [28]. The lower yield of singlet oxygen by photosensitized FMN relative to RF may be attributed to its phosphate group. This group is negatively charged under aqueous conditions at pH 7–8 and can form strong hydrogen bonds and electrostatic interactions with surrounding solvent molecules. These interactions affect the distribution and dynamics of excited states, reducing triplet state formation and lifetime and lowering the efficiency of singlet oxygen production [28]. Conversely, RF is devoid of a phosphate group, resulting in diminished hydrogen bonding and electrostatic effects, and enabling more efficient intersystem crossing and triplet state maintenance. The reported triplet quantum yields are approximately 0.375 for RF and 0.225–0.30 for FMN at pH 8 [29]. Thus, the phosphate group present in FMN facilitates solvent interactions that suppress triplet state dynamics, resulting in reduced singlet oxygen yields relative to RF. Despite the presence of an isoalloxazine ring, LF exhibited considerably reduced photosensitizing property in comparison to RF, FAD, and FMN. This phenomenon is attributed to the absence of a complete ribityl side chain in LF, a structural element that is integral to the photoreactions within RF, contributing to its degradation and photosensitizing properties.

It is important to note that classical singlet oxygen quantum yield values reported for RF, lumiflavin, and lumichrome were obtained almost exclusively under UV excitation (e.g., 337 nm N_2_ laser, 355 nm Neodymium-doped Yttrium Aluminum Garnet (Nd:YAG) laser, or 365 nm mercury lamps) [27,30,31,32]. Under these conditions, lumichrome and lumiflavin exhibit substantially higher absorption efficiency and consequently even higher quantum yields than RF. However, these UV-driven photophysical characteristics cannot be extrapolated to visible light irradiation, where the absorption profiles and excited state dynamics of flavins differ from their UV-excited behavior. The differences observed among flavin derivatives in this study reflect their relative photosensitizing properties under a controlled experimental framework designed for applied comparison. Since this assay does not measure singlet oxygen quantum yields or intrinsic photophysical parameters, a direct comparison with literature values obtained under different excitation wavelengths and experimental conditions is beyond the scope of this study.

Conversely, our systematic analysis of the photostability of DPBF under multiple light sources reveals the mechanistic limitations of the classical, absorbance-based DPBF method. These limitations have not been documented previously. Supported by cross-validation with different probes, the integration of DPBF fluorescence detection is expected to establish a robust methodological framework for evaluating visible light-induced photosensitization that does not rely on UV-dependent quantum yield benchmarks.

### 2.6. Comparison of the Photosensitizing Properties with ABDA

Another probe often used for detecting singlet oxygen is 9,10-anthracenediyl-bis(methylene)dimalonic acid (ABDA). ABDA has been reported to be less sensitive than DPBF for assessing singlet oxygen, but as a water-soluble probe, it can measure the extent of singlet oxygen generation in aqueous environments [11]. In the subsequent experiment, the singlet oxygen levels generated via type II photosensitization reactions of RF and its derivatives, the water-soluble photosensitizers, were measured using ABDA. The objective of this cross-validation with two different probes (DPBF and ABDA) was to ensure data reliability and compare photosensitizing property across various solvent conditions.

**Figure 6:**
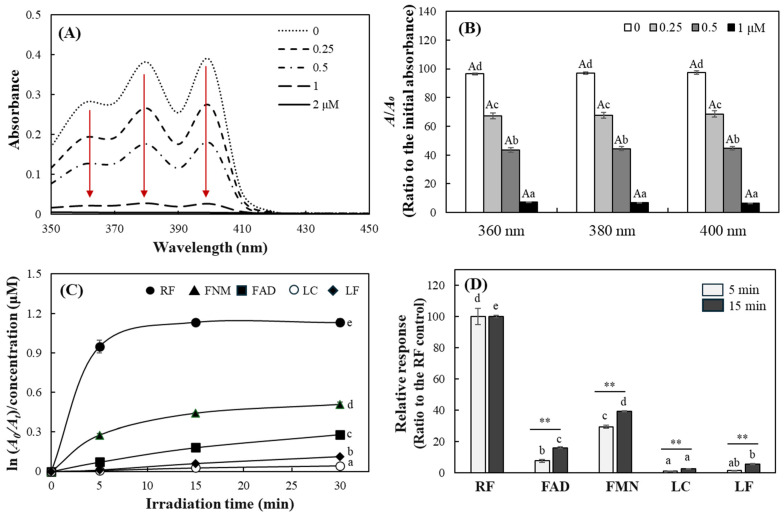
Evaluation of photosensitizing properties of RF and its derivatives using ABDA probe under the blue LED. Different concentrations of RF were incubated with ABDA under the blue LED (10 W/m^2^) for 5 min (**A**), and the relative changes in ABDA absorbance at three peaks were compared (**B**). The concentration-adjusted singlet oxygen levels generated from RF and its derivatives under 10 W/m^2^ blue LED were evaluated according to the ABDA absorbance changes at 400 nm for 30 min (**C**) and the relative ABDA responses based on RF of 100 at 5 and 15 min irradiation were also calculated (**D**). Each value represents the mean ± SD (*n* = 3). Different upper– and lower–case letters indicate a significant difference (*p* < 0.05) among different measurement peaks and among the same concentrations of RF, respectively, based on one-way ANOVA and Tukey’s HSD test (in (**B**)). Different letters indicate significant difference (*p* < 0.05) (in (**C**,**D**)). ** significantly different between 5 and 15 min according to Student’s *t*-test (**, *p* < 0.01 in (**D**)).

ABDA exhibits three major absorbance peaks within the range of 350–450 nm. Exposure to blue LED irradiation (10 W/m^2^) with various RF concentrations for 5 min resulted in a concentration-dependent decrease in absorbance at all three peaks (Figure 6A,B). A comparison of the decrease in absorbance across different RF concentrations revealed similar patterns at all three peak wavelengths (Figure 6B). Consequently, 400 nm was selected as the representative wavelength for ABDA in subsequent analyses to facilitate comparisons among RF and its derivatives. The results also showed that RF exhibited the highest changes in ABDA response, followed by FMN and FAD, with production increasing over longer irradiation times. In contrast, LC and LF produced much smaller changes in ABDA responses (Figure 6C). This trend was consistent with the results obtained under organic solvent conditions of EtOH using DPBF.

A comparison of the changes over different light exposure times revealed that the order of potency remained constant. However, RF showed saturated activity following 5 min of irradiation, with a minimal subsequent increase in ABDA responses, whereas the other derivatives continued to increase steadily up to 30 min of light exposure (Figure 6C). Therefore, when comparing relative singlet oxygen production based on RF, the other derivatives exhibited an increase in relative activity over time (Figure 6D); their activity correction considering the entire reaction time was not possible, in contrast to the assay using DPBF. This is because the reactivity of ABDA with singlet oxygen is considerably lower than that of DPBF, requiring high-intensity (10 W/m^2^) blue LED irradiation and a sufficient light exposure time of over 5 min for measuring the activity of other derivatives except RF. This aspect is considered a limitation of the use of ABDA compared to DPBF in measuring the activity of light-sensitive photosensitizers. A notable finding is that FMN generated relative singlet oxygen levels of 29.2 and 39.0 at 5 and 15 min of irradiation, respectively, in the assay using ABDA, as compared to 100 of RF (Figure 6D), while it exhibited relative activities of 8.398 and 8.591 in absorbance- and fluorescence-based measurements using DPBF, respectively (Table 2). This phenomenon can be attributed to the structural characteristics of FMN, which contains a phosphate moiety and exhibits strong hydrophilicity. Consequently, its photosensitizing property is expressed more effectively in an aqueous condition compared to an EtOH environment.

In photosensitizing reactions, singlet oxygen generated via the type II mechanism is known to be strongly influenced by the physicochemical properties of the surrounding medium. Factors such as the solubility of the photosensitizer, solvent polarity, dissolved oxygen content, and singlet oxygen lifetime can differ markedly between hydrophilic and hydrophobic environments, thereby leading to changes in the efficiency of singlet oxygen production [33]. Therefore, an appropriate probe that considers these diverse factors is necessary to measure and compare photosensitizing property. This study reports that measuring the singlet oxygen production of RF and its derivatives under light based on the fluorescence response of DPBF effectively represents their type II photosensitizing property. This method allows for precise measurement of photosensitizing property under low light intensity and short exposure times. In summary, this work does not propose a fundamentally new photochemical method. Instead, it demonstrates how an established DPBF-based assay can be practically applied for relative comparison of photosensitizing activity under visible-light conditions, where conventional quantum-yield-based approaches are limited. This application-oriented use is expected to be useful in applied fields such as food chemistry and visible-light flavin photochemistry.

## 3. Materials and Methods

### 3.1. Experimental Materials

DPBF (97%), RB, RF, LC, ABDA and dimethyl sulfoxide (DMSO) were purchased from Sigma-Aldrich Co. (St. Louis, MO, USA). FMN sodium salt (95.8%), FAD disodium salt (>93%), and LF were obtained from Santa Cruz Biotechnology, Inc. (Dallas, TX, USA). EtOH was from Duksan Pure Chemical (Ansan, Republic of Korea). DPBF was dissolved in EtOH, whereas all other reagents were dissolved in DMSO, aliquoted, and stored at −80 °C until use.

### 3.2. Light Irradiation System

Three types of LED irradiation devices (Bissol LED Inc., Seoul, Republic of Korea) and a fluorescent light (FL; ARS-3300OL, Aris Co., Seoul, Republic of Korea) were used in this study. The peak wavelength (λpeak) and dominant wavelength (λd) of the fluorescent light were 545 and 492 nm, respectively (Figure 4C). The λ peak values for the For the red, green, and blue LED sources were 656, 518, and 447 nm, respectively, and the λd were 641, 524, and 452 nm, respectively (Figure 4C). The irradiance (W/m^2^) of each light source was measured and adjusted using a solar power meter (TM-207, TENMARS, Taipei, Taiwan) in all experiments [34].

### 3.3. Absorption and Fluorescence Properties of DPBF and Photosensitizers

To analyze the optical properties of DPBF and different photosensitizers used in this study, each compound was diluted to a final concentration of 100 μM in EtOH, and 200 μL aliquots were dispensed into a 96-well plate. The absorbance and fluorescence spectra were measured using a microplate reader (SpectraMax M2; Molecular Devices, Sunnyvale, CA, USA). Absorbance spectra were recorded from 350 to 700 nm, and fluorescence emission spectra were obtained with excitation at 350 nm under non-cutoff conditions [35].

### 3.4. Analysis of DPBF Photostability

The photostability of DPBF was evaluated under red, green, and blue LED light sources and a fluorescent light. DPBF was diluted to 100 μM in EtOH, with 200 μL of the solution being added to each well of a 96-well plate. The diluted DPBF solutions were incubated under the different light sources or in the dark during 60 min. Changes in absorbance at 410 nm and fluorescence at excitation/emission = 350/480 nm (non-cutoff) were monitored at each time point. Photostability was calculated by all values to the initial intensity, set at 100%. The half-life of DPBF degradation was calculated using linear regression analysis in the range showing a linear decrease. All experiments were conducted at a temperature of 25 °C. The experimental setup involved the application of red and green LED and fluorescent light irradiations at a constant intensity of 10 W/m^2^, while blue LED irradiations were conducted at both 0.5 W/m^2^ and 10 W/m^2^ intensities.

### 3.5. Detection of Relative Levels of Singlet Oxygen Generated by Photosensitization

The relative levels of singlet oxygen generated from photosensitizers upon light irradiation was quantitatively measured using DPBF as a singlet oxygen probe. All photosensitizers were diluted in EtOH to a final concentration range of 0–4 μM with the final vehicle concentration maintained at below 1%, except for LC and LF, which were diluted to a concentrations range of 0–100 μM. DPBF was also diluted in EtOH to a final concentration of 100 μM and mixed with each photosensitizer solution at a 1:1 ratio. The samples were then incubated at 25 °C in the dark or under 0.5 W/m^2^ blue LED irradiation for up to 5 min. Absorbance at 410 nm and fluorescence at excitation/emission = 350/480 nm were measured every 1 min using a microplate reader (SpectraMax M2). The values were normalized to the control group, and the amount of singlet oxygen produced was calculated using Equation (1).

Relative singlet oxygen level generated = ln (A_0_/A_t_) (1)

A_0_ = The color or fluorescence intensity of DPBF at time 0.

Aₜ = The color or fluorescence intensity of DPBF at time t.

The relative levels (RL) of DPBF responses made by each photosensitizer were compared based on the concentration-adjusted level, RL = {ln (A_0_/A_t_)/concentration (μM)}. The relative photosensitizing activity of RF and its derivatives was also calculated based on the slope of the RL vs. irradiation time plot [d{ln (A_0_/A_t_)/concentration (μM)}/dt].

Photosensitizing responses in an aqueous phase was evaluated using ABDA. ABDA (400 μM) solution was mixed with each photosensitizer at a 1:1 ratio and irradiated with a 10 W/m^2^ blue LED for 30 min, during which the absorption spectra were measured in the range of 350–450 nm. Changes in the ABDA absorbance of three peaks at 360, 380, and 400 nm in the presence of different concentrations of RF were measured during 5 min irradiation. Additionally, the concentration-adjusted relative level {ln (A_0_/A_t_)/concentration (μM)} was calculated using the change in ABDA absorbance at 400 nm, and the relative activity of each derivative was compared relative to RF (100).

### 3.6. Statistical Analysis

All values represent the mean ± standard deviation (SD). Each experiment was repeated 3–12 times. Statistical significance was evaluated using Student’s *t*-test and one-way ANOVA in SPSS program (IBM SPSS Statistics 29, SPSS Inc. Chicago, IL, USA) with Tukey’s honestly significant difference (HSD) test (*p* < 0.05) for comparing multiple results.

## 4. Conclusions

In the present study, the type II photosensitizing property of different photosensitizers, including RF and its related heterocyclic flavin derivatives using DPBF, was quantitatively evaluated. RB, a representative type II photosensitizer, produced singlet oxygen in a concentration- and irradiation time-dependent manner, which was consistent with both absorbance- and fluorescence-based detection. In particular, DPBF fluorescence exhibited a strong correlation with RB concentration, confirming the suitability of this method for evaluating the photosensitizing property.

DPBF exhibited the least stability under blue LEDs, which have emission wavelengths close to its maximum absorption; relatively higher stability was observed under red and green LEDs. The relative singlet oxygen levels generated from RF and its derivatives were analyzed using DPBF under blue LED irradiation. There is overlap between RF or its derivatives and DPBF absorption, which causes interference in absorbance-based assays, and fluorescence analysis significantly minimized this issue. Furthermore, fluorescence-based detection was found to be more sensitive and precise than absorbance measurement. It maintained high linearity and low variation in measurements among different sample concentrations even with short irradiation times and low irradiation intensity, thereby establishing it as a more reliable approach for evaluating the type II photosensitizing property. Among the tested compounds, RF exhibited the highest photosensitizing property, followed by FMN, FAD, LF, and LC. This trend was consistent with the results obtained from both absorbance- and fluorescence- based measurements using DPBF in an organic solvent, as well as with the results obtained from ABDA in an aqueous condition.

In summary, this study demonstrates that DPBF fluorescence-based detection is a more sensitive and accurate method than traditional absorbance-based approaches for evaluating singlet oxygen-induced responses. It can effectively overcome spectral interference from colored photosensitizers and deliver reliable results in real sample analyses. Therefore, this method is expected to serve as a tool for the precise comparison and characterization of the type II photosensitizing property across a wide range of compounds.

## Figures and Tables

**Table 1:** Quantitative DPBF responses induced by different concentrations of RF and its derivatives under different periods of blue LED irradiation based on absorbance and fluorescence changes in DPBF.

		Absorbance					Fluorescence				
Irradiation Time (min)		1	2	3	4	5	1	2	3	4	5
0		0.00 ± 0.03 ^a(1)^	0.00 ± 0.03 ^a^	0.00 ± 0.03 ^a^	0.00 ± 0.03 ^a^	0.00 ± 0.04 ^a^	0.00 ± 0.02 ^a^	0.00 ± 0.02 ^a^	0.00 ± 0.03 ^a^	0.00 ± 0.03 ^a^	0.00 ± 0.03 ^a^
RF (μM)	0.25	0.17 ± 0.03 ^b^	0.32 ± 0.04 ^b^	0.45 ± 0.05 ^b^	0.56 ± 0.06 ^b^	0.65 ± 0.07 ^b^	0.20 ± 0.03 ^b^	0.35 ± 0.04 ^b^	0.54 ± 0.05 ^b^	0.70 ± 0.05 ^ab^	0.86 ± 0.06 ^ab^*
	0.5	0.31 ± 0.03 ^bc^	0.61 ± 0.05 ^bc^	0.85 ± 0.05 ^bc^	1.02 ± 0.05 ^b^	1.15 ± 0.06 ^b^	0.35 ± 0.03 ^b^	0.68 ± 0.04 ^c^	1.04 ± 0.05 ^c^*	1.37 ± 0.05 ^bc^*	1.72 ± 0.06 ^bc^*
	1	0.61 ± 0.05 ^cd^	1.14 ± 0.07 ^cd^	1.49 ± 0.09 ^d^	1.66 ± 0.12 ^c^	1.68 ± 0.21 ^c^	0.63 ± 0.05 ^b^	1.34 ± 0.06 ^d^*	2.08 ± 0.06 ^d^**	2.78 ± 0.06 ^d^**	3.54 ± 0.07 ^d^*
	2	1.09 ± 0.11 ^e^	1.84 ± 0.19 ^e^	2.02 ± 0.31 ^e^	1.95 ± 0.54 ^cd^	1.76 ± 0.81 ^c^	1.24 ± 0.05 ^c^*^(2)^	2.70 ± 0.05 ^e^**	4.20 ± 0.01 ^e^**	5.68 ± 0.01 ^e^**	6.55 ± 0.02 ^e^**
	4	1.67 ± 0.18 ^f^	2.30 ± 0.03 ^f^	2.24 ± 1.13 ^e^	2.07 ± 2.26 ^d^	1.91 ± 0.69 ^c^	2.12 ± 0.22 ^d^*	4.64 ± 0.34 ^f^**	5.94 ± 0.36 ^f^**	6.20 ± 0.37 ^f^**	6.79 ± 0.35 ^f^**
FAD (μM)	0.25	0.00 ± 0.01 ^a^	0.01 ± 0.01 ^b^	0.02 ± 0.01 ^b^	0.03 ± 0.01 ^a^	0.04 ± 0.02 ^a^	0.02 ± 0.01 ^a^	0.02 ± 0.00 ^a^	0.03 ± 0.01 ^a^	0.04 ± 0.01 ^a^	0.05 ± 0.01 ^a^
	0.5	−0.01 ± 0.01 ^a^	0.01 ± 0.01 ^b^	0.02 ± 0.01 ^b^	0.04 ± 0.01 ^b^	0.06 ± 0.01 ^b^	0.03 ± 0.01 ^b^	0.05 ± 0.00 ^b^*	0.08 ± 0.01 ^b^**	0.10 ± 0.01 ^b^**	0.13 ± 0.01 ^b^**
	1	−0.03 ± 0.02 ^a^	0.04 ± 0.01 ^c^	0.08 ± 0.01 ^c^	0.12 ± 0.01 ^c^	0.15 ± 0.01 ^c^	0.06 ± 0.01 ^c^	0.11 ± 0.01 ^c^	0.17 ± 0.02 ^c^*	0.22 ± 0.01 ^c^**	0.27 ± 0.02 ^c^**
	2	0.08 ± 0.04 ^b^	0.11 ± 0.02 ^d^	0.19 ± 0.02 ^d^	0.25 ± 0.01 ^d^	0.31 ± 0.02 ^d^	0.11 ± 0.01 ^d^	0.22 ± 0.01 ^d^**	0.34 ± 0.02 ^d^**	0.45 ± 0.02 ^d^**	0.54 ± 0.03 ^d^**
	4	0.17 ± 0.03 ^c^	0.27 ± 0.03 ^e^	0.41 ± 0.02 ^e^	0.53 ± 0.02 ^e^	0.62 ± 0.01 ^e^	0.21 ± 0.02 ^e^	0.42 ± 0.01 ^e^**	0.63 ± 0.02 ^e^**	0.85 ± 0.01 ^e^**	1.06 ± 0.02 ^e^**
FMN (μM)	0.25	0.02 ± 0.01 ^a^	0.01 ± 0.03 ^a^	0.02 ± 0.04 ^a^	0.04 ± 0.04 ^a^	0.05 ± 0.04 ^a^	0.02 ± 0.04 ^a^	0.03 ± 0.04 ^a^	0.04 ± 0.04 ^a^	0.05 ± 0.05 ^a^	0.06 ± 0.05 ^a^
	0.5	−0.02 ± 0.01 ^a^	−0.01 ± 0.04 ^a^	0.00 ± 0.04 ^a^	0.02 ± 0.05 ^a^	0.03 ± 0.05 ^a^	0.03 ± 0.04 ^a^	0.05 ± 0.04 ^a^	0.08 ± 0.04 ^ab^	0.09 ± 0.05 ^ab^	0.12 ± 0.05 ^ab^
	1	0.00 ± 0.06 ^a^	0.06 ± 0.05 ^ab^	0.10 ± 0.06 ^a^	0.13 ± 0.07 ^a^	0.15 ± 0.07 ^a^	0.08 ± 0.04 ^a^	0.15 ± 0.04 ^a^	0.20 ± 0.04 ^b^	0.26 ± 0.05 ^b^	0.33 ± 0.06 ^b^
	2	0.06 ± 0.00 ^b^	0.19 ± 0.05 ^b^	0.28 ± 0.06 ^b^	0.35 ± 0.07 ^b^	0.39 ± 0.08 ^b^	0.14 ± 0.04 ^b^	0.31 ± 0.05 ^b^	0.45 ± 0.05 ^c^	0.57 ± 0.06 ^c^	0.71 ± 0.07 ^c^*
	4	0.20 ± 0.02 ^c^	0.35 ± 0.06 ^c^	0.60 ± 0.07 ^c^	0.72 ± 0.06 ^c^	0.77 ± 0.07 ^c^	0.28 ± 0.07 ^c^	0.65 ± 0.08 ^c^	1.00 ± 0.09 ^d^*	1.32 ± 0.10 ^d^**	1.66 ± 0.12 ^d^**
LC (μM)	6.25	0.01 ± 0.04 ^a^	0.03 ± 0.03 ^a^	0.05 ± 0.03 ^a^	0.06 ± 0.04 ^a^	0.08 ± 0.04 ^a^	0.03 ± 0.02 ^a^	0.03 ± 0.01 ^a^	0.04 ± 0.03 ^b^	0.06 ± 0.04 ^b^	0.08 ± 0.04 ^b^
	12.5	0.01 ± 0.03 ^a^	0.05 ± 0.03 ^a^	0.08 ± 0.03 ^ab^	0.11 ± 0.04 ^ab^	0.11 ± 0.04 ^ab^	0.03 ± 0.02 ^a^	0.04 ± 0.01 ^ab^	0.06 ± 0.03 ^b^	0.08 ± 0.04 ^b^	0.10 ± 0.04 ^b^
	25	−0.02 ± 0.01 ^a^	−0.01 ± 0.01 ^a^	0.01 ± 0.02 ^a^	0.04 ± 0.02 ^a^	0.05 ± 0.02 ^a^	0.05 ± 0.01 ^a^**	0.05 ± 0.0 ^ab^**	0.09 ± 0.02 ^b^**	0.12 ± 0.02 ^b^**	0.14 ± 0.02 ^b^**
	50	−0.09 ± 0.01 ^a^	−0.05 ± 0.04 ^a^	0.00 ± 0.03 ^a^	0.03 ± 0.04 ^a^	0.06 ± 0.05 ^a^	0.07 ± 0.05 ^a^**	0.09 ± 0.04 ^b^**	0.16 ± 0.04 ^c^**	0.21 ± 0.04 ^c^**	0.25 ± 0.05 ^c^**
	100	0.00 ± 0.02 ^a^	0.05 ± 0.04 ^a^	0.11 ± 0.05 ^b^	0.16 ± 0.06 ^b^	0.22 ± 0.07 ^b^	0.12 ± 0.02 ^b^**	0.19 ± 0.02 ^c^**	0.29 ± 0.03 ^d^**	0.35 ± 0.04 ^d^**	0.42 ± 0.05 ^d^**
LF (μM)	6.25	0.04 ± 0.01 ^a^	0.04 ± 0.01 ^a^	0.05 ± 0.01 ^a^	0.06 ± 0.01 ^a^	0.08 ± 0.01 ^b^	0.00 ± 0.01 ^b^	0.04 ± 0.01 ^b^	0.02 ± 0.01 ^b^	0.04 ± 0.01 ^b^	0.05 ± 0.02 ^b^
	12.5	0.03 ± 0.02 ^a^	0.06 ± 0.01 ^a^	0.08 ± 0.01 ^b^	0.12 ± 0.01 ^b^	0.15 ± 0.01 ^c^	0.03 ± 0.01 ^b^	0.08 ± 0.00 ^b^	0.09 ± 0.01 ^b^	0.13 ± 0.02 ^b^	0.15 ± 0.02 ^b^
	25	−0.03 ± 0.02 ^a^	0.03 ± 0.01 ^a^	0.08 ± 0.02 ^b^	0.14 ± 0.02 ^b^	0.19 ± 0.02 ^d^	0.09 ± 0.01 ^b^	0.14 ± 0.02 ^b^	0.21 ± 0.01 ^c^	0.29 ± 0.02 ^c^**	0.36 ± 0.02 ^c^*
	50	−0.02 ± 0.07 ^a^	0.10 ± 0.01 ^b^	0.22 ± 0.03 ^c^	0.32 ± 0.03 ^c^	0.41 ± 0.03 ^e^	0.14 ± 0.04 ^c^	0.31 ± 0.03 ^c^	0.47 ± 0.04 ^d^	0.62 ± 0.04 ^d^*	0.79 ± 0.04 ^d^**
	100	0.18 ± 0.03 ^b^	0.38 ± 0.04 ^c^	0.62 ± 0.03 ^d^	0.79 ± 0.03 ^d^	0.90 ± 0.03 ^f^	0.28 ± 0.04 ^d^	0.60 ± 0.05 ^d^**	0.94 ± 0.05 ^e^**	1.28 ± 0.05 ^e^**	**1.64 ± 0.06 ^e^**

^(1)^ Each value represents the mean ± SD (*n* = 3). Different letters indicate a significant difference among different concentrations including 0 control of each compound. ^(2)^ *, ** significantly different (**bold**) from values of absorbance-based analysis according to Student’s *t*–test (*, *p* < 0.05; **, *p* < 0.01).

**Table 2:** Comparison of photosensitizing properties adjusted by concentrations of Rb and its derivatives under blue LED irradiation and their relative activity quantified by the absorbance- or fluorescence-based method.

		Absorbance					Fluorescence				
Irradiation Time (min)		1	2	3	4	5	1	2	3	4	5
	Average ^(1)^	0.573	1.029	1.503	1.985	2.190	0.655	1.322	2.099	2.792	3.421
	SD ^(2)^	0.098	0.290	0.354	0.300	0.464	0.099	0.094	0.033	0.042	0.108
RF	CV ^(3)^ (%)	17.13	28.17	23.57	15.11	21.18	15.17	7.08	1.56	1.50	3.16
	RL ^(4)^	n/c ^(5)^	100	100	100	100	n/c	100	100	100	100
	^(6)^ R^2^ = 0.992; ^(7)^ Slope = 0.419; ^(8)^ RA = 100.0						R^2^ = 0.999; Slope = 0.700; RA = 100.0				
	Average	0.007	0.043	0.078	0.115	0.147	0.058	0.105	0.152	0.206	0.250
	SD	0.033	0.022	0.024	0.017	0.020	0.004	0.007	0.022	0.026	0.033
FAD	CV (%)	491.21	51.34	31.02	14.92	13.44	6.92	6.57	14.35	12.57	13.24
	RL	n/c	4.15	5.20	5.78	6.70	n/c	7.98	7.25	7.37	7.32
	R^2^ = 1.000; Slope = 0.035; RA = 8.394						R^2^ = 1.000; Slope = 0.049; RA = 6.930				
	Average	0.025	0.052	0.098	0.136	0.159	0.075	0.137	0.198	0.253	0.318
	SD	0.045	0.050	0.057	0.056	0.057	0.009	0.026	0.040	0.059	0.074
FMN	CV (%)	183.23	94.38	57.75	40.83	36.01	12.59	18.69	20.09	23.32	23.13
	RL	n/c	5.10	6.53	6.85	7.25	n/c	10.37	9.45	9.07	9.29
	R^2^ = 0.995; Slope = 0.035; RA = 8.398						R^2^ = 1.000; Slope = 0.060; RA = 8.591				
	Average	0.000	0.002	0.003	0.005	0.006	0.002	0.004	0.006	0.007	0.009
	SD	0.001	0.001	0.002	0.003	0.003	0.001	0.000	0.001	0.002	0.002
LC	CV (%)	174.85	54.10	55.78	63.04	57.89	9.58	11.19	12.70	23.58	27.03
	RL	n/c	0.18	0.22	0.24	0.26	n/c	0.33	0.28	0.27	0.25
	R^2^ = 0.996; Slope = 0.001; RA = 0.324						R^2^ = 0.996; Slope = 0.002; RA = 0.226				
	Average	0.002	0.003	0.005	0.007	0.009	0.003	0.006	0.008	0.012	0.015
	SD	0.003	0.002	0.002	0.002	0.002	0.000	0.000	0.001	0.001	0.002
LF	CV (%)	161.57	55.76	30.02	23.78	20.60	10.68	4.45	14.07	10.41	12.17
	RL	n/c	0.33	0.34	0.37	0.42	n/c	0.45	0.40	0.42	0.43
	R^2^ = 0.999; Slope = 0.002; RA = 0.445						R^2^ = 0.999; Slope = 0.003; RA = 0.417				

^(1)^ Average; the mean values of concentration-adjusted levels {ln (A_0_/A_t_)/concentration (μM)} of DPBF responses at different concentrations (0.25–4 or 6.25–100 μM) of photosensitizers at indicated irradiation time periods. ^(2)^ SD; standard deviation of each average value (*n* = 3–5) ^(3)^ CV; coefficient of variation (%) = SD/average. ^(4)^ RL; the relative levels of DPBF responses based on RF-induced changes (100) at each irradiation time point. ^(5)^ n/c; not calculated. ^(6)^ R^2^; correlation coefficients between relative levels of DPBF responses and irradiation time. ^(7)^ Slope; d{ln (A_0_/A_t_)/concentration (μM)}/dt. ^(8)^ RA; the relative activity calculated based on the slope of Rb (concentration-adjusted level vs. irradiation time) under blue LED.

## Data Availability

The original contributions presented in this study are included in this article. Further inquiries can be directed to the corresponding author.

## Supplementary Materials

The following supporting information can be downloaded at: https://www.mdpi.com/article/10.3390/ijms27010066/s1.
