# Supplementary material for: A Comparative Analysis of Absorbance- and Fluorescence-Based 1,3-Diphenylisobenzofuran Assay and Its Application for Evaluating Type II Photosensitization of Flavin Derivatives
Source: Int J Mol Sci. 2025 Dec 20;27(1):66. doi: 10.3390/ijms27010066 (PMC12786022; doi:10.3390/ijms27010066)
Supplement: Supplementary file 1 [file ijms-27-00066-s001.zip › ijms-4010044-supplementary.pdf]

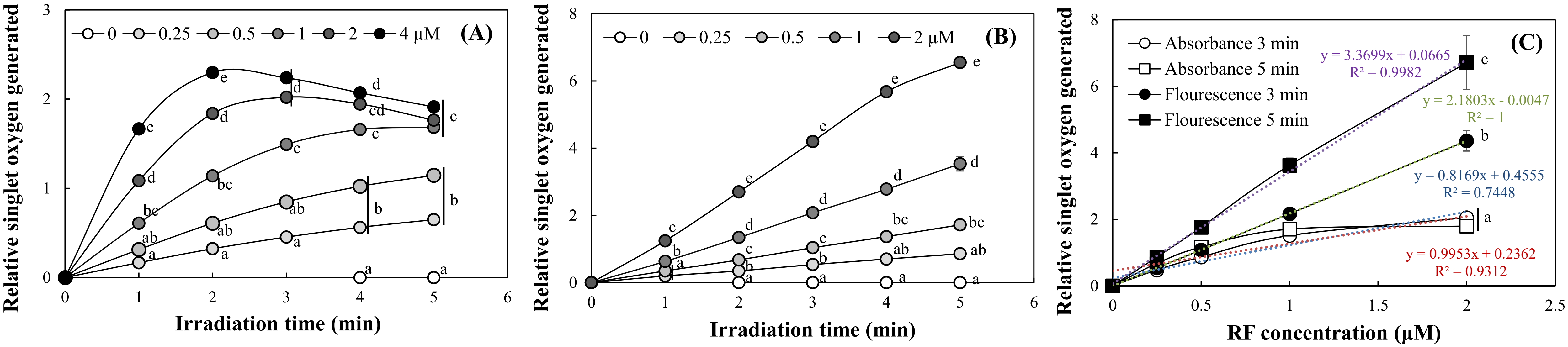

(Supplementary Figure) Fig. S1. Comparison of singlet oxygen level generated by RF under blue LED using DPBF. Changes in color (A) and emission fluorescence (B) intensities of DPBF were monitored under the blue LED with RF for 5 min, and the relative levels {ln (A_0/A_t)} of singlet oxygen generated in each condition were calculated. A correlation between the relative singlet oxygen levels calculated based on absorbance or fluorescence changes and RF concentrations was also performed (C). Each value represents the mean ± SD (n=3-12). Different letters indicate significant difference ($p < 0.05$) based on one way ANOVA.
